# Intravoxel Incoherent Motion (IVIM) Diffusion-Weighted Imaging (DWI) in Patients with Liver Dysfunction of Chronic Viral Hepatitis: Segmental Heterogeneity and Relationship with Child-Turcotte-Pugh Class at 3 Tesla

**DOI:** 10.1155/2018/2983725

**Published:** 2018-12-16

**Authors:** Lei Ding, Lianxiang Xiao, Xiangtao Lin, Chunmei Xiong, Lingbo Lin, Shijun Chen

**Affiliations:** ^1^Shandong University, Department of Infectious Diseases, Jinan Central Hospital Affiliated to Shandong University, Jinan, 250021 Shandong Province, China; ^2^Shandong Medical Imaging Research Institute, Jinan, 250021 Shandong Province, China; ^3^Shandong University, Jinan Infectious Diseases Hospital, Jinan, 250021 Shandong Province, China; ^4^Jinan Infectious Diseases Hospital, Jinan, 250021 Shandong Province, China

## Abstract

**Background:**

Few studies focused on the region of interest- (ROI-) related heterogeneity of liver intravoxel incoherent motion (IVIM) diffusion-weighted imaging (DWI). The aim of the study was to evaluate the differences of liver IVIM parameters among liver segments in cirrhotic livers (chronic viral hepatitis).

**Material and Methods:**

This was a retrospective study of 82 consecutive patients with chronic liver disease who underwent MRI examination at the Jinan Infectious Diseases Hospital between January 2015 and December 2016. IVIM DWI (seven different *b* values) was performed on a Siemens 3.0-T MRI scanner. Pure molecular diffusion (*D*), pseudodiffusion (*D*^∗^), and perfusion fraction (*f*) in different liver segments were evaluated.

**Results:**

*f*, D, and *D*^∗^ were different among the liver segments (all *p* < 0.05), indicating heterogeneity in IVIM parameters among liver segments. *f* was consistently higher in Child-Turcotte-Pugh (CTP) class A compared with CTP class B + C (*p* < 0.01). *D* and *D*^∗^ were higher in CTP class A compared with CTP class B + C (*p* < 0.05). In patients with mean *f* value of >0.29, the AUC was 0.88 (95% CI: 0.81-0.96), with 86.8% sensitivity and 81.8% specificity for predicting CTP class A from CTP class B + C.

**Conclusion:**

Liver IVIM could be a promising method for classifying the severity of segmental liver dysfunction of chronic viral hepatitis as evaluated by the CTP class, which provides a noninvasive alternative for evaluating segmental liver dysfunction with accurate selection of ROIs. Potentially it can be used to monitor the progression of CLD and LC in the future.

## 1. Introduction

Liver function estimation plays an essential role in predicting the prognosis of patients with chronic liver disease (CLD) or liver cirrhosis (LC), both of which ultimately lead to liver failure. For patients within a background of CLD or LC with or without hepatocellular carcinoma (HCC), assessment of liver function is also an integral part of the therapeutic decision making and can help physicians make the appropriate treatment decision [1, 2]. In clinical practice, indocyanine green clearance test, elastography, and clinical scoring systems such as the Child-Turcotte-Pugh (CTP) or Model for End-Stage Liver Disease (MELD) scores are used to evaluate whole liver function. CTP score is a widely used and validated predictor of long-term survival in CLD and LC, and patients are grouped into class A, B, and C according to the total score of 5-6, 7-9, and 10-15, respectively [3].

Magnetic resonance imaging (MRI) using gadoxetic acid and intravoxel incoherent motion (IVIM) diffusion-weighted imaging (DWI) has recently shown a potential for the evaluation of segmental liver dysfunction [4–8]. IVIM DWI, which was initially described by Le Bihan and Turner [9] in brain imaging, has the potential to measure both true molecular diffusion and the incoherent motion of water molecules in the capillary network. By using the IVIM model and multiple sufficiently low *b* values (<200 mm^2^/sec), not only can pure diffusion characteristics (*D*) be separated from pseudodiffusion caused by microscopic circulation in tissue, but perfusion characteristics (pseudodiffusion coefficient (*D*^∗^)) and their proportion (perfusion fraction (*f*)) can also be derived [10–12]. Using IVIM DWI, perfusion and diffusion factors can be separated [2]. Nevertheless, for CLD or LC, the perfusion and microscopic phenomena of liver are heterogeneous due to progressive increase in connective tissue and reduced liver perfusion [4, 9]. Indeed, the variations associated with the acquisition sites of shear-wave elastography for evaluating liver fibrosis stage have been proven by Samir et al. [13]. Thus, the IVIM parameters may vary among different segments due to the regions of interest (ROIs) location. Recent studies of liver dysfunction or fibrosis evaluated by IVIM refer to different ROIs location and boundary from a single segment to the whole liver [7, 14–18].

Few studies focused on the ROI-related heterogeneity of liver IVIM parameters. Therefore, the aim of this study was to evaluate the differences of liver IVIM parameters among liver segments in cirrhotic livers caused by chronic viral hepatitis and determine the relationships between IVIM measurements and liver dysfunction assessment according to the CTP scoring system.

## 2. Material and Methods

### 2.1. Study Design

This was a retrospective study of 82 consecutive patients with CLD who underwent MRI examination at the Department of Radiology of Jinan Infectious Diseases Hospital between January 2015 and December 2016. The study was approved by the ethics committee of Jinan Infectious Diseases Hospital. The need for individual consent was waived because of the retrospective nature of the study.

### 2.2. Patients

For those patients, MRI examination was primarily performed to observe the morphological changes of liver and the secondary changes of hepatitis/cirrhosis, such as nodules and ascites, and to exclude HCC. The inclusion criteria were (1) >18 years of age, (2) chronic infection with hepatitis B virus (HBV) or hepatitis C virus (HCV), and (3) IVIM was performed. The exclusion criteria were (1) previously received local treatment for liver disease, (2) unable to complete the entire MR imaging examination, (3) other diffuse liver disease (primary sclerosing cholangitis, primary biliary cirrhosis, hepatic adipose infiltration, etc.), (4) HCC confirmed by MRI, cyst, and hemangioma 1 cm or greater in diameter confirmed by MRI (other tumors or tumor-like lesions were not found for those patients), (5) portal vein emboli, or (6) alcohol abuse or alcoholic cirrhosis.

### 2.3. Biochemical Tests and Liver Function

All patients underwent serological tests in the same laboratory within 1 week before or after MRI. The severity of liver disease was estimated by the CTP scoring system.

### 2.4. MRI Examination and IVIM Parameters

All patients were instructed to fast and abstain from food and water overnight prior to MRI examination. MRI was performed using a 3.0-T scanner (Magnetom Verio, Siemens Healthcare, Erlangen, Germany). A body coil served as the transmitter and a 6-element spine matrix coil in combination with the body matrix were used as the receiver. At first, coronal T2-weighted imaging (half-Fourier acquisition single-shot turbo spin-echo (HASTE), repetition time (TR)/echo time (TE) 1400/86 ms, flip angle 10, matrix 512 × 512, field of view 400 × 400 mm, slice thickness 5 mm, 20% gap, 30 slices) was performed.

The transverse MRI protocol included liver dome scout-triggered transverse T2-weighted turbo spin-echo sequence (TR/TE 4251/105 ms, matrix 560, field of view 400 × 400 mm, slice thickness 5 mm, 20% gap, 30 slices), and 3D in-phase and out-of-phase breath-hold fast spoiled gradient-echo imaging (TR/TE, 4.0/2.5 and 1.2 ms, flip angle 10, matrix 512 × 512, field of view 450 × 390 mm, slice thickness 3 mm, 72 slices).

Free-breathing, IVIM DWI was performed using a single-shot spin-echo echo planar sequence (SE-EPI), with gradient reversal fat suppression (TR/TE 6500/67 ms, echo spacing 0.52 ms, FOV 400 × 262 mm, using 7 *b* values of 0, 50, 100, 150, 200, 400, and 800 s/mm^2^).

### 2.5. Image Analysis

**Figure 1:**
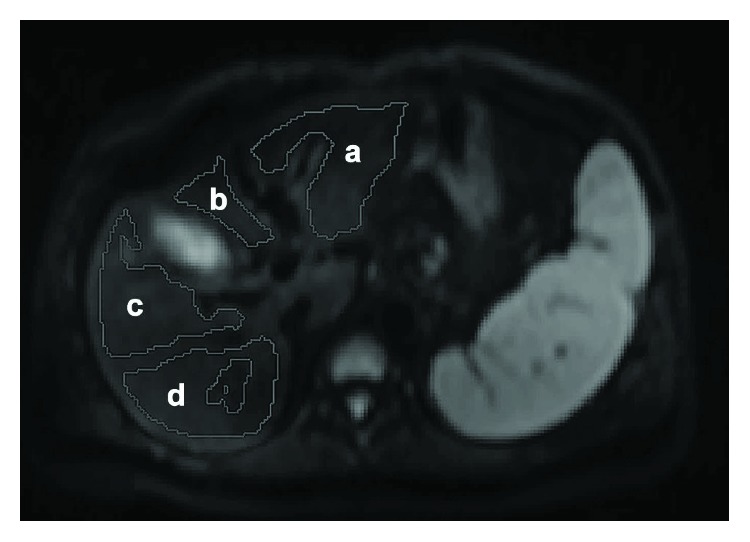
Regions of interest (ROIs) drawing for intravoxel incoherent motion diffusion-weighted imaging measurements. a = extra segment of the left lobe; b = medial segment of the left lobe; c = anterior segment of the right lobe; d = posterior segment of the right lobe.

Postprocessing of the IVIM data were performed by using the MITK diffusion software (developed by the German Cancer Research Center, Division of Medical and Biological Informatics, Heidelberg, Germany) to acquire IVIM parameters of *f*, *D*, and *D*^∗^. For the liver parenchyma, four irregular ROIs (designed to carefully preserve at least 5 mm to the edge of the liver, including whole segments as large as possible and excluding visible vessels, focal hepatic lesions such as cyst and hemangioma, or imaging artifacts) were placed by choosing different levels of the liver (slices near the visceral and diaphragmatic surfaces were discarded to eliminate intestinal gas and respiratory motion artifacts). The ROIs were manually drawn in the extra segments of the left lobe (EL), medial segments of the left lobe (ML), anterior segments of the right lobe (AR), and posterior segments of the right lobe (PR) (Figure 1). All ROIs were positioned on DWI with *b* values of 50 by two radiologists, one with 15 years (XTL) and the other with 7 years (LXX) of experience in abdominal MRI. The two radiologists were blind to the clinical characteristics of the patients. Interobserver agreement for all IVIM parameters was excellent, with Cronbach's *α* of 0.951 for *f*, 0.876 for *D*, and 0.861 for *D*^∗^.

For each liver segment, IVIM parameters (*f*, *D*, and *D*^∗^) were calculated by the average of measured value at 6-15 different level of transverse liver sections. The average *f*, *D*, and *D*^∗^ values of the four liver segments were taken as the whole liver IVIM parameters.

### 2.6. Statistical Analysis

Continuous data were tested for normal distribution using the Kolmogorov-Smirnov test. The IVIM parameters were expressed as mean ± standard deviations. One-way ANOVA with the LSD post hoc test was used to evaluate IVIM parameters among different liver segments. IVIM parameters between the CTP class A group and the CTP class B + C group were compared using the Student *t*-test. The receiver operator characteristic curve (ROC) was used to compare the ability of *f*, *D*, and *D*^∗^ values in discriminating patients with CTP class A and CTP class B + C. Multiple linear regression analysis was used to evaluate the correlations between the IVIM parameters and the CTP scores. All statistical analyses were performed using SPSS 21.0 for Windows (IBM, Armonk, NY, USA). Two-sided *p* values < 0.05 were considered statistically significant.

## 3. Results

### 3.1. Patients

**Figure 2:**
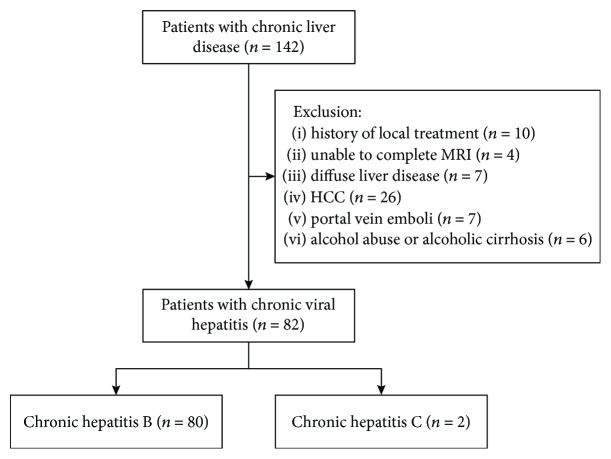
Patient flowchart.

A total of 142 patients with CLD were considered for inclusion. Patients were excluded if they had previously received a local treatment for liver disease (*n* = 10), if they were unable to complete the entire MRI examination (*n* = 4), if they had other diffuse liver disease (*n* = 7), if they had HCC confirmed by MRI (*n* = 26), if they had portal vein emboli (*n* = 7), or if they had alcohol abuse or alcoholic cirrhosis (*n* = 6). As shown in Figure 2, 82 patients (age range 24-77 years) with chronic viral hepatitis were included in this study. The clinical characteristics of these patients are listed in Table 1.

### 3.2. IVIM Parameters in Different Location

**Figure 3:**
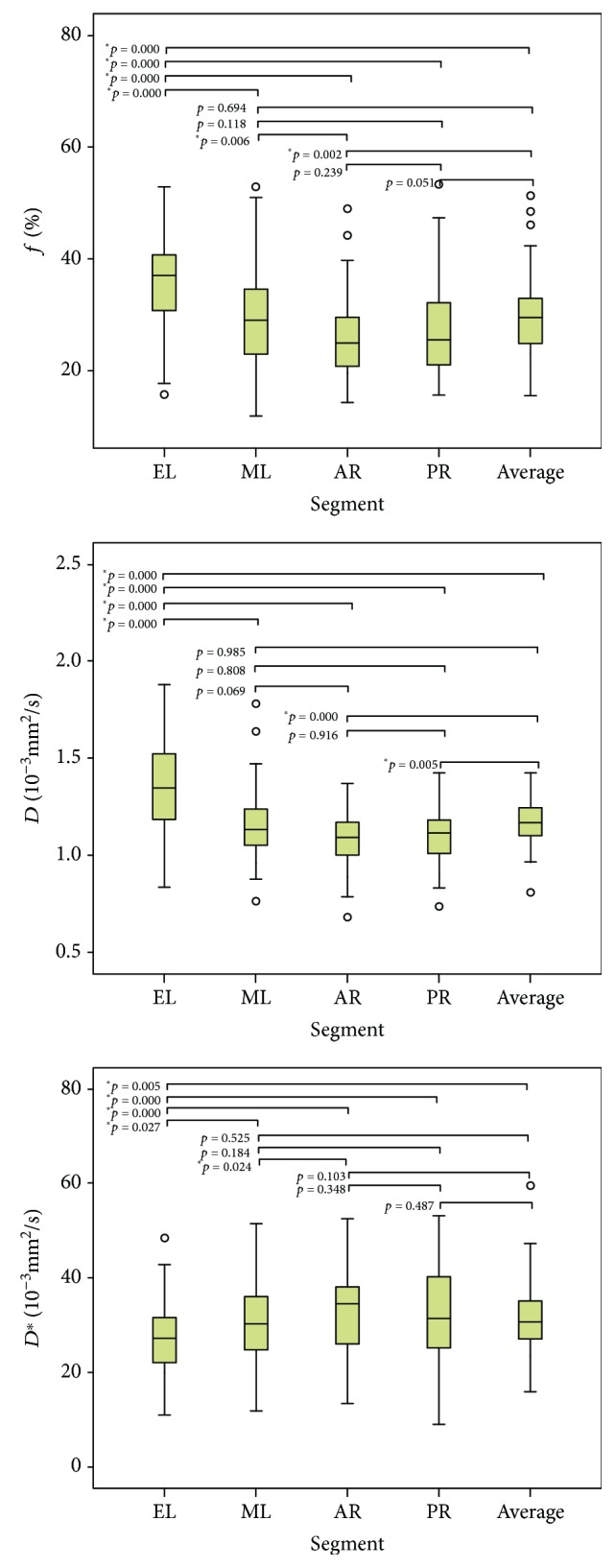
Box plots of *f*, *D*, and *D*^∗^ in different liver segments.

The IVIM parameters of the different liver segments and of the whole liver were normally distributed (*p* = 0.310 − 0.899, Kolmogorov-Smirnov test). The descriptive statistics are presented in Table 2.  Figure 3 shows that *f*, *D*, and *D*^∗^ were significantly different among the liver segments, indicating heterogeneity in IVIM parameters among different liver segments.

### 3.3. Relationship between IVIM Parameters and CTP Class

Table 3 shows *f*, *D*, and *D*^∗^ of different liver segments according to the CTP class. *f* was consistently higher in CTP class A than in CTP class B + C (all *p* < 0.01). *D* was higher in CTP class A compared with CTP class B + C in the EL, PR, and whole liver (all *p* < 0.05). *D*^∗^ was higher in CTP class A compared with CTP class B + C in the EL, ML, PR, and whole liver (all *p* < 0.05).

### 3.4. ROC Analysis

**Figure 4:**
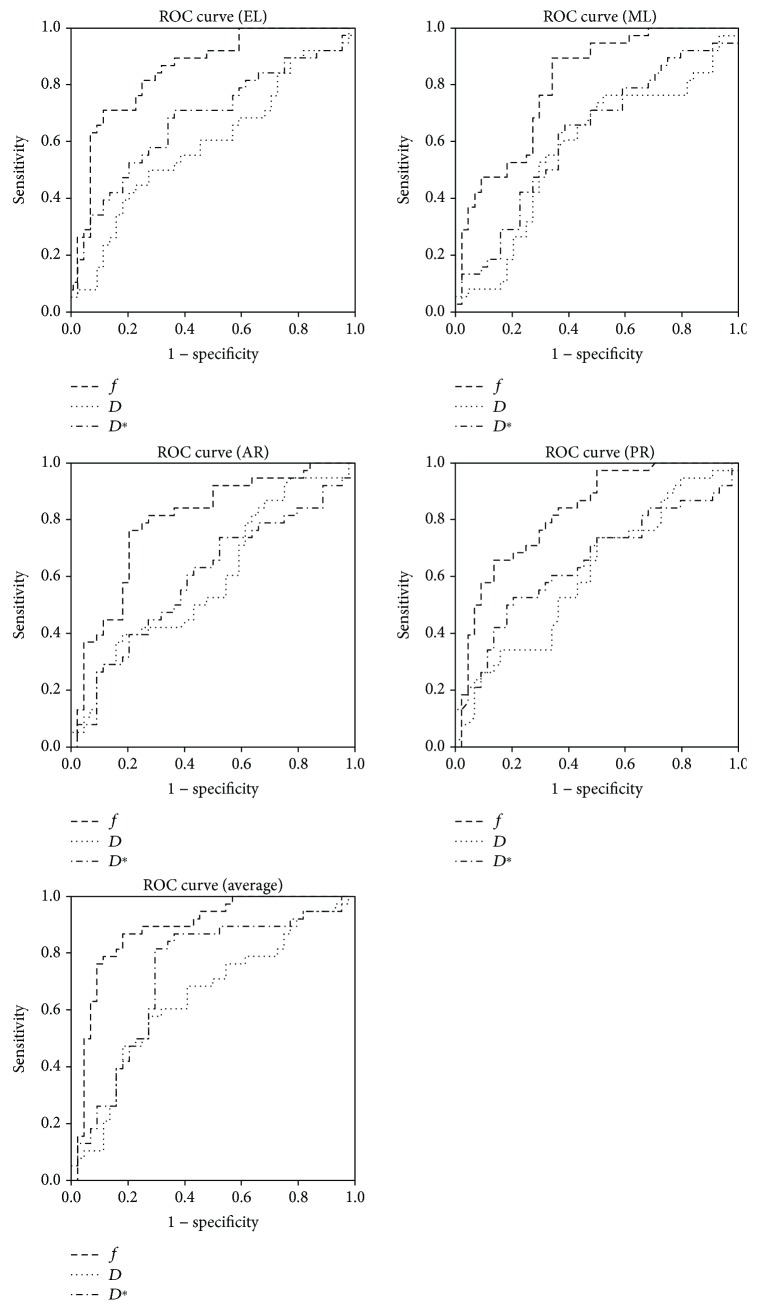
Receiver operating characteristic curves comparing *f*, *D*, and *D*^∗^ in different segments as predictors of CTP class (A vs. B + C).

As shown in Figure 4, the area under the ROC curves (AUC) for *f*, *D*, and *D*^∗^ value were statistically significant. In patients with mean *f* value of >0.29, the AUC was 0.88 (95% CI: 0.81-0.96), which was the highest of the three IVIM parameter, leading to 86.8% sensitivity and 81.8% specificity for predicting CTP class A from CTP class B + C (Table 4).

### 3.5. Multivariate Analysis

Multiple linear regression analysis showed that in viral hepatitis patients, *f* (*p* < 0.001) and *D* (*p* = 0.038) were independently associated with the CTP class, while *D*^∗^ was not associated (*p* = 0.451).

### 3.6. Typical Cases

**Figure 5:**
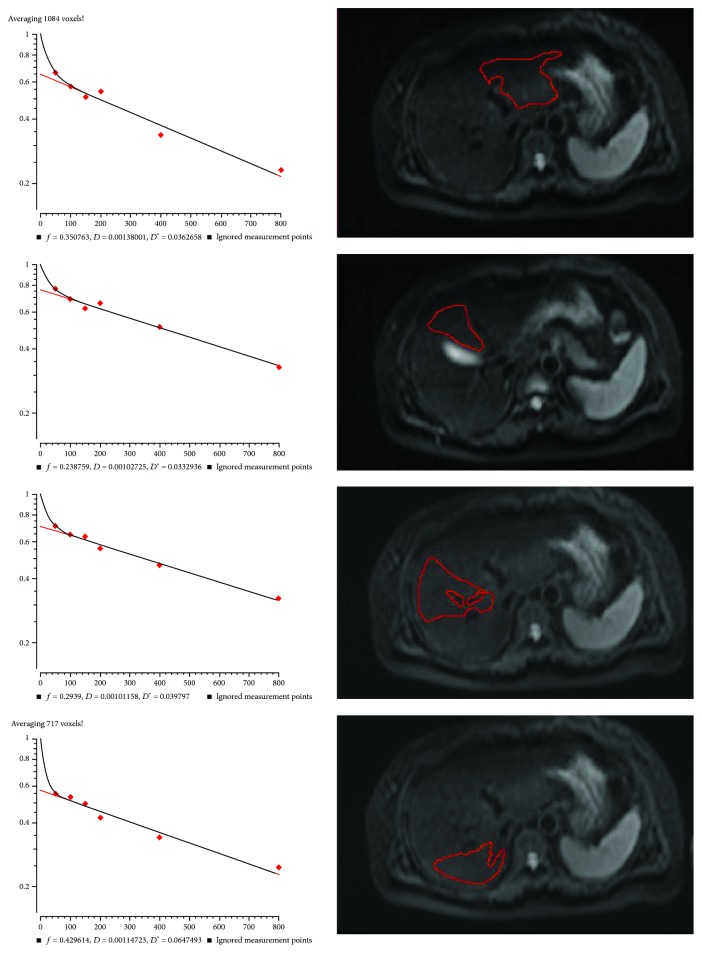
IVIM measurements in different liver segments in a 52-year-old female with chronic hepatitis B, CTP score of 6 (class A).

**Figure 6:**
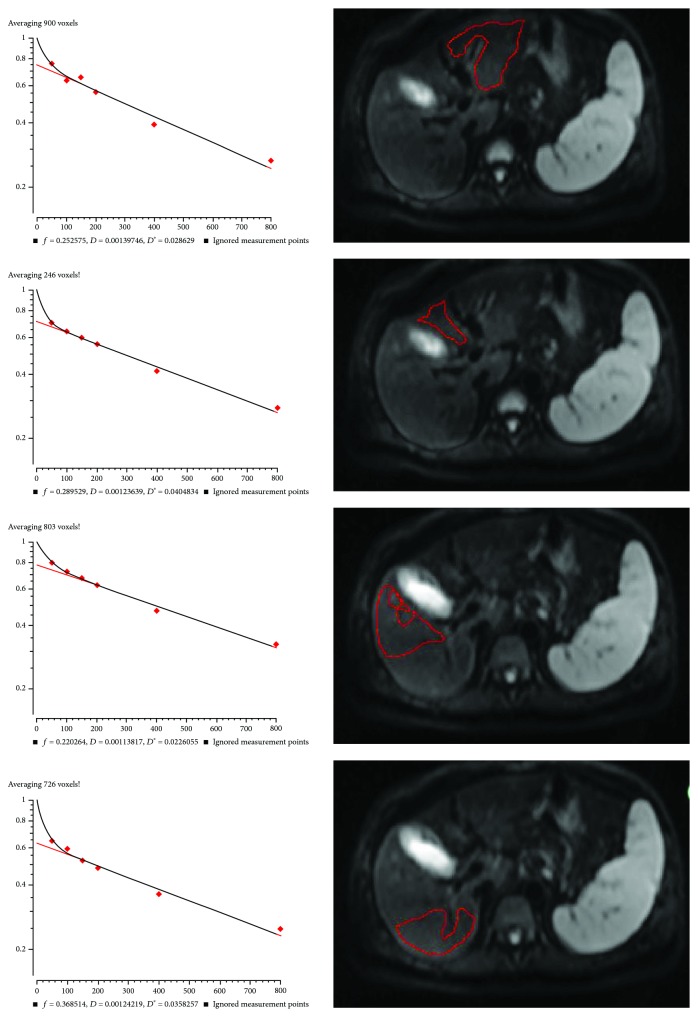
IVIM measurements in different liver segment in a 67-year-old female with chronic hepatitis B, CTP score of 9 (class B).

Figures 5 and 6 present two typical cases. Both cases were relatively stable. The patient presented in Figure 5 was CTP class A. *f* EL was 0.35, *f* ML was 0.24, *f* AR was 0.29, *f* PR was 0.43, and whole liver *f* was 0.33. According to the critical value of 0.29 in the present study, the liver function evaluated by *f* value showed that the whole liver *f* value was consistent with CTP class A, but if a single ROI is placed in the left inner lobe or right anterior lobe, or if using multiple ROIs, a false positive result would be obtained and the patient would be identified as a CTP class B + C. In a similar manner, in Figure 6, the patient was CTP class B. *f* EL was 0.25, *f* ML was 0.29, *f* AR was 0.22, *f* PR was 0.37, and whole liver *f* was 0.28. Nevertheless, if the ROI was placed in the right posterior lobe, then the patient would be determined as CTP class A. These two typical cases clearly demonstrate the importance of liver heterogeneity and liver function local assessment in cirrhotic patients.

## 4. Discussion

The present study revealed a significant variability of IVIM parameters among different liver segments. There were statistically significant higher *f* and *D* values, but lower *D*^∗^ values in EL compared with the other segments, as supported by a study by Dijkstra et al. [5]. The heterogeneity of *f* values was partly similar to that observed in previous studies [4, 5]. Nevertheless, the present study suggests location dependency in all IVIM parameters including the microperfusion component *D*^∗^ and the pure molecular diffusion component *D*. For the heterogeneity of *D*^∗^, the present study is consistent with the study by Dijkstra et al. [5], but both studies contradict Luciani et al. [4]. A number of studies may be responsible for these differences, including the types of pathologies, ethnic groups, and genetics. In addition, the range observed in the present study was lower than what they reported [4]. This may reflect the use of different *b* values calculation methods since we chose the three parameters fit model on the MITK diffusion workstation. Significant heterogeneity of *D* between segments was not observed in these two studies, which could be due to the relatively small number of patients or mild changes in the microenvironment in healthy individuals.

Furthermore, a previous study showed obvious variation, and a large range of *f* values, *D* values, and *D*^∗^ values for F0 stage liver tissue [2]. It is well accepted that liver cirrhosis is associated with reduced liver perfusion, particularly with reduced portal flow [19–21]. In an experimental study on rats using perfusion computed tomography (CT), the relative blood flow in the left lobe was 17% higher than in the right lobe of the liver [22]. This is also supported by Su et al. [23] in a study of hepatic perfusion by dual-source CT. They found that the hepatic perfusion index (HPI) was significantly higher in segment 3 (extra left lobe) than in segments 5 to 8 (right lobe) and suggested that this might be related to the anatomy of the liver vessels. This is supported by the compensatory increase of the left lobe in liver cirrhosis.

We believe that besides the histological changes such as fat and iron content and technical difference such as the choice of *b* values and cardiac or respiratory artifacts, the acquisition site of ROIs also has an important influence explaining, at least in part, the large range of reported IVIM parameters. These issues add to the heterogeneity observed among liver segments, further complicating the interpretation of the results.

Reduced liver perfusion and progressive increased connective tissue are considered to be the possible mechanisms that could underlie the reduction of IVIM parameters in CLD and liver cancer [24, 25]. The *D* values reflect both intra- and extracellular molecular diffusion, while *D*^∗^ and *f* values reflect the microcirculation. It has been reported that *f* values are decreased with the increasing severity of the necroinflammatory activity [4, 26–33]. Therefore, IVIM parameter changes may reflect not only the fibrosis degree and perfusion changes but also the hepatitis activity such as inflammatory infiltration, hepatic cell edema, and cholestasis. In the present study, the *f*, *D*, and *D*^∗^ values were all decreased with increasing CTP score, which is supported by a previous study by Zhang et al. [34]. According to the multiple linear regression analysis, the *f* and *D* values were independently associated with the CTP score, but not *D*^∗^. This could partially be because *D*^∗^ is not a well reproducible measure influenced by liver fibrosis [35]. A moderate relation was found between the average *f* value and CTP score, and a mild relation between either average *D* value or CTP score. The IVIM parameters of CTP class A were significantly higher than that of CTP class B + C, yet there was only mild to moderate correlation between the IVIM parameters and CTP score. This can be partially due to the distribution of the patients, mostly CTP score of 5 to 7, as observed in most patients of the present study. Furthermore, the *D* values were influenced by confounders such as fibrosis, fat, and iron, which commonly coexist with liver disease.

Based on previous studies and ours, IVIM DWI could be a quick and repeatable noninvasive MR modality that enables qualitative and quantitative evaluation of tissue diffusivity. It could potentially become a reliable imaging modality to quantify changes in CLD. One of the advantage of IVIM DWI is that it can be integrated into routine abdominal MRI sequences. Compared with the use of gadolinium chelates, there is no restriction of abnormal renal function, as the impaired renal function leads to increased hepatobiliary excretion after injection of Gd-EOB-DTPA [36, 37].

The present study has several limitations. First, histopathological confirmation of liver fibrosis stage was not performed. Secondly, there was no patient with normal liver as a negative control group. In addition, there was only a few patients with high CTP score. Nevertheless, the changes of IVIM parameters in the CTP A group and the CTP B + C group still reflected the tendency of negative correlation with liver dysfunction. In addition, because of software limitations on the MRI system, it was not possible to acquire any data of *D*^∗^ between *b* = 0 and 50 s/mm^2^, which may result in the relative lower *D*^∗^ measurement in the present study as well as in others' [5–7, 38–41]. Although the obvious variation and poor reproducibility of IVIM parameters were doubted by some study [35], it still showed a reasonable potential for quantifying CLD. Among the most influential factors, segmental dependency-related heterogeneity may be underestimated in previous studies.

## 5. Conclusions

IVIM DWI imaging of the liver is a promising modality for classifying the severity of liver dysfunction of chronic viral hepatitis as evaluated by CTP class. It provides a noninvasive alternative for evaluating segmental liver dysfunction. Clinically, we can potentially use IVIM to monitor the progression of CLD and LC in the future. The heterogeneity of IVIM measurements should be considered for the choice of ROIs. Further research is warranted regarding the value of IVIM MR imaging in the diagnosis and staging of CLD and LC.

## Figures and Tables

**Table 1:** Clinical characteristics of 82 patients with chronic viral hepatitis evaluated using IVIM MRI.

Characteristics	Value (mean, SD)	Median (range)
Age (years)	52.7 ± 11.9	51 (27-77)
Male/female, *n* (%)	51 (62.2)/31 (37.8)	
BMI (kg/m^2^)	24.1 ± 2.8	24.2 (17.9-24.3)
Hepatitis B/hepatitis C	80/2	
INR	1.3 ± 0.3	1.26 (0.95-2.9)
ALT (IU/L)	81.4 (141)	40.5 (13-1005)
AST (IU/L)	92.7 ± 105	54.5 (19-691)
ALB (g/L)	34.4 ± 6.5	34 (17-54.7)
TBIL (mmol/L)	40.9 ± 62.1	21.7 (4.9-375.4)
AFP (ng/mL)	119.4 ± 250.6	14.9 (0.84->2000)
CTP score, mean or n	6.9 ± 1.4	
CTP class A (5~6)	40	
5	24	
6	16	
CTP class B (7~9)	40	
7	29	
8	5	
9	6	
CTP class C (10)	2	

BMI: body mass index; INR: international normalized ratio; ALT: alanine transaminase; AST: aspartate transaminase; ALB: albumin; TBIL: total bilirubin; AFP: *α*-fetoprotein.

**Table 2:** IVIM DWI parameters among the liver segments.

Liver segment	*f* (%)		*D* (10^−3^ mm^2^/s)		*D* ^∗^ (10^−3^ mm^2^/s)	
	Mean ± SD	95% CI	Mean ± SD	95% CI	Mean ± SD	95% CI
EL	36.14 ± 7.80	34.42-37.85	1.34 ± 0.23	1.29-1.39	27.58 ± 7.79	25.86-29.29
ML	29.22 ± 8.67	27.32-31.12	1.15 ± 0.16	1.11-1.18	30.41 ± 7.96	28.66-32.16
AR	25.94 ± 6.60	24.49–-27.39	1.09 ± 0.13	1.06-1.11	33.31 ± 7.89	31.58-35.05
PR	27.35 ± 8.00	25.59-29.10	1.10 ± 0.12	1.07-1.13	32.11 ± 9.90	29.94-34.28
Whole	29.69 ± 7.04	28.14-31.24	1.17 ± 0.12	1.15-1.19	31.22 ± 7.13	29.65-32.79
*p*	<0.001		<0.001		<0.001	

EL: extra segment of the left lobe; ML: medial segment of the left lobe; AR: anterior segment of the right lobe; PR: posterior segment of the right lobe.

**Table 3:** *f*, *D*, and *D*^∗^ of different liver segments according to the CTP class.

Liver segment	*f* (%)			*D* (10^−3^ mm^2^/s)			*D* ^∗^ (10^−3^ mm^2^/s)		
	CTP A	CTP B + C	Sig.	CTP A	CTP B + C	Sig.	CTP A	CTP B + C	Sig.
EL	38.53 ± 7.07	33.76 ± 8.00	0.006	1.41 ± 0.22	1.27 ± 0.22	0.006	29.58 ± 7.04	25.93 ± 8.07	0.034
ML	32.35 ± 7.63	26.20 ± 8.76	0.001	1.18 ± 0.13	1.12 ± 0.12	0.114	32.68 ± 7.46	28.17 ± 8.07	0.011
AR	28.67 ± 6.19	23.61 ± 6.75	0.001	1.11 ± 0.10	1.08 ± 0.14	0.303	34.70 ± 8.45	31.98 ± 7.17	0.119
PR	29.90 ± 7.82	24.86 ± 7.64	0.005	1.14 ± 0.12	1.08 ± 0.12	0.023	34.27 ± 10.1	30.05 ± 9.35	0.038
Whole	32.36 ± 6.21	27.11 ± 7.06	0.001	1.21 ± 0.10	1.13 ± 0.12	0.005	33.56 ± 7.22	28.99 ± 6.36	0.003
*n*	40	42		40	42		40	42	

EL: extra segment of the left lobe; ML: medial segment of the left lobe; AR: anterior segment of the right lobe; PR: posterior segment of the right lobe.

**Table 4:** ROC curves data of *f*, *D*, and *D*^∗^ for distinguishing CTP class A vs. B + C.

Segment	AUC		
	*f* (*p* value)	*D* (*p* value)	*D* ^∗^ (*p* value)
EL	0.85 (<0.001)	0.59 (0.158)	0.68 (0.005)
ML	0.80 (<0.001)	0.58 (0.234)	0.62 (0.062)
AR	0.79 (<0.001)	0.59 (0.142)	0.60 (0.139)
PR	0.83 (<0.001)	0.61 (0.099)	0.65 (0.021)
Whole liver	0.88 (<0.001)	0.65 (0.024)	0.73 (<0.001)

EL: extra segment of the left lobe; ML: medial segment of the left lobe; AR: anterior segment of the right lobe; PR: posterior segment of the right lobe.

## Data Availability

The datasets generated during the current study are available from the corresponding author on reasonable request.
